# Absence of self-reported neuropsychiatric and somatic symptoms after Omicron variant SARS-CoV-2 breakthrough infections

**DOI:** 10.1093/braincomms/fcad092

**Published:** 2023-03-25

**Authors:** Marcel S Woo, Christina Mayer, Thomas Theo Brehm, Gabriele Andersen, Angelika Weigel, Bernd Löwe, Ansgar W Lohse, Marylyn M Addo, Christian Gerloff, Johannes K M Knobloch, Julian Schulze zur Wiesch, Manuel A Friese

**Affiliations:** Institute of Neuroimmunology and Multiple Sclerosis, University Medical Center Hamburg-Eppendorf, Hamburg 20251, Germany; Institute of Neuroimmunology and Multiple Sclerosis, University Medical Center Hamburg-Eppendorf, Hamburg 20251, Germany; German Center for Infection Research (DZIF), Partner Site Hamburg-Lübeck-Borstel-Riems, Hamburg 20246, Germany; I. Department of Medicine, University Medical Center Hamburg-Eppendorf, Hamburg 20246, Germany; Department of Occupational Safety and Health, University Medical Center Hamburg-Eppendorf, Hamburg 20246, Germany; Department of Psychosomatic Medicine and Psychotherapy, University Medical Center Hamburg-Eppendorf, Hamburg 20246, Germany; Department of Psychosomatic Medicine and Psychotherapy, University Medical Center Hamburg-Eppendorf, Hamburg 20246, Germany; German Center for Infection Research (DZIF), Partner Site Hamburg-Lübeck-Borstel-Riems, Hamburg 20246, Germany; I. Department of Medicine, University Medical Center Hamburg-Eppendorf, Hamburg 20246, Germany; German Center for Infection Research (DZIF), Partner Site Hamburg-Lübeck-Borstel-Riems, Hamburg 20246, Germany; I. Department of Medicine, University Medical Center Hamburg-Eppendorf, Hamburg 20246, Germany; Institute for Infection Research and Vaccine Development, University Medical Center Hamburg-Eppendorf, Hamburg 20246, Germany; Department of Neurology, University Medical Center Hamburg-Eppendorf, Hamburg 20251, Germany; German Center for Infection Research (DZIF), Partner Site Hamburg-Lübeck-Borstel-Riems, Hamburg 20246, Germany; Institute of Medical Microbiology, Virology and Hygiene, Department for Infection Prevention and Control, University Medical Center Hamburg-Eppendorf, Hamburg 20246, Germany; German Center for Infection Research (DZIF), Partner Site Hamburg-Lübeck-Borstel-Riems, Hamburg 20246, Germany; I. Department of Medicine, University Medical Center Hamburg-Eppendorf, Hamburg 20246, Germany; Institute of Neuroimmunology and Multiple Sclerosis, University Medical Center Hamburg-Eppendorf, Hamburg 20251, Germany

**Keywords:** SARS-CoV-2 breakthrough infection, Omicron, Long COVID, neuropsychiatric deficits

## Abstract

Persistent somatic and neuropsychiatric symptoms have been frequently described in patients after infection with severe acute respiratory syndrome coronavirus 2 even after a benign clinical course of the acute infection during the early phases of the coronavirus severe acute respiratory syndrome coronavirus 2 pandemic and are part of Long COVID. The Omicron variant emerged in November 2021 and has rapidly become predominant due to its high infectivity and suboptimal vaccine cross-protection. The frequency of neuropsychiatric post-acute sequelae after infection with the severe acute respiratory syndrome coronavirus 2 Omicron and adequate vaccination status is not known. Here, we aimed to characterize post-acute symptoms in individuals with asymptomatic or mildly symptomatic breakthrough infection with severe acute respiratory syndrome coronavirus 2. These individuals had either proven infection with the Omicron variant (*n* = 157) or their infection occurred in 2022 where Omicron was the predominant variant of severe acute respiratory syndrome coronavirus 2 in Germany (*n* = 107). This monocentric cross-sectional study was conducted at the University Medical Center Hamburg-Eppendorf between 11 February 2022 and 11 April 2022. We employed questionnaires addressing self-reported somatic symptom burden (Somatic Symptom Scale 8) and neuropsychiatric symptoms including mood (Patient Health Questionnaire 2), anxiety (Generalized Anxiety Disorder 7), attention (Mindful Attention Awareness Scale) and fatigue (Fatigue Assessment Scale) in a cohort of hospital workers. Scores were compared between 175 individuals less than 4 weeks after positive testing for severe acute respiratory syndrome coronavirus 2, 88 individuals more than 4 weeks after positive testing and 87 severe acute respiratory syndrome coronavirus 2 uninfected controls. The majority (*n* = 313; 89.5%) of included individuals were vaccinated at least three times. After recovery from infection, no significant differences in scores assessing neuropsychiatric and somatic symptoms were detected between the three groups (severe acute respiratory syndrome coronavirus 2 uninfected controls, individuals less and more than 4 weeks after positive testing) independent of age, sex, preconditions and vaccination status. In addition, self-reported symptom burden did not significantly correlate with the number of vaccinations against severe acute respiratory syndrome coronavirus 2, time from recovery or the number of infections. Notably, in all three groups, the mean scores for each item of our questionnaire lay below the pathological threshold. Our data show that persistent neuropsychiatric and somatic symptoms after recovery from severe acute respiratory syndrome coronavirus 2 infection in fully vaccinated hospital workers do not occur more frequently than that in uninfected individuals. This will guide healthcare professionals in the clinical management of patients after recovery from breakthrough infections with severe acute respiratory syndrome coronavirus 2.

## Teaser

Woo and Mayer *et al*. investigated self-reported somatic and neuropsychiatric symptoms in a cross-sectional study of a closely monitored cohort of hospital workers after infection with the severe acute respiratory syndrome coronavirus 2 Omicron variant in comparison with uninfected individuals. No differences were detected between groups adjusted for age, sex, pre-existing conditions and vaccination status.

## Graphical Abstract

**Graphical Abstract:**
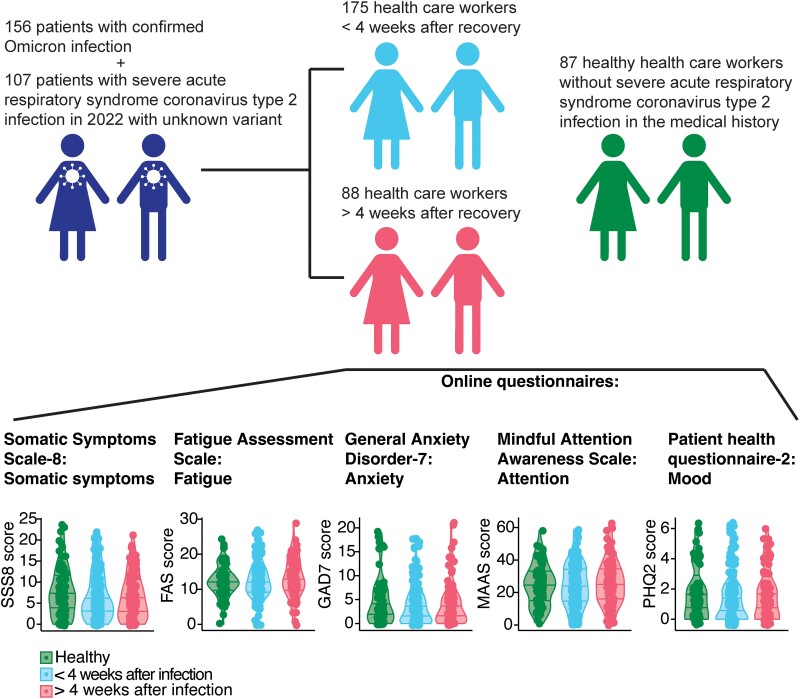

## Introduction

Increasing numbers of individuals worldwide contracted an infection with severe acute respiratory syndrome coronavirus 2 (SARS-CoV-2), systematic analysis, diagnosis and treatment of potential post-acute symptoms and complications are of crucial importance.^1,2^ Neuropsychiatric symptoms following acute infection can cause a high individual burden of disease^3^ and socio-economical costs due to the enormous number of people who experienced an infection.

Long COVID has been used as an umbrella term to describe the multitude of persistent symptoms attributed to long-term effects of a SARS-CoV-2 infection. These symptoms can affect nearly every organ system and include symptoms such as fatigue, headaches, shortness of breath, fever or cognitive impairment.^4^ Currently, there is no clear consensus regarding the definition of post-acute sequelae. Most commonly, the term Long COVID is used when symptoms persist for more than 4 weeks after the infection and cannot be explained by other causes.^5,6^ During the first wave of the coronavirus SARS-CoV-2 (COVID-19) pandemic, when vaccines were not yet available, it has been estimated that 31–69% of patients suffered from post-acute sequelae.^7-9^ Certain risk factors have been identified during the acute disease in recent observational and retrospective cohort studies that might predict the development of Long COVID. These include type 2 diabetes, SARS-CoV-2 RNAemia, Epstein–Barr virus viraemia and specific autoantibodies.^10,11^ Furthermore, female sex, high body mass index (BMI), social deprivation, advanced age, the presence of multiple comorbidities, a severe disease course and being unvaccinated predisposed to Long COVID.^5,12^ Neurological deficits during acute COVID-19 range from unspecific dizziness, headache and nausea to acute encephalitis and cerebral ischaemia.^13-15^ Neuropsychiatric symptoms of Long COVID remain ill-defined due to unspecific clinical presentation and diagnostic findings. For individuals infected during the first waves of COVID-19 in 2020 and 2021, persistent neuropsychiatric deficits were associated with severe acute disease and pre-existing mental health conditions.^16,17^ However, several studies also described prolonged neuropsychiatric deficits after mild SARS-CoV-2 infection in previously healthy individuals.^18,19^

Due to the availability of vaccination, an increasing number of SARS-CoV-2 infections occur in vaccinated individuals and are referred to as breakthrough infections. The frequency and severity of post-COVID-19 symptoms in these breakthrough infections are still unclear. A recent study that utilized the US Department of Veterans Affairs national healthcare databases and compared the medical health records of more than 33 000 breakthrough infections with more than 6 million controls showed that vaccinations reduce the risk of post-acute death and Long COVID.^20^ However, vaccination only conferred partial protection since the incidence of post-acute sequelae was still elevated in comparison with healthy control groups.^20^ This is supported by other studies^21^ that observed only partial protection of vaccination against persistent symptoms. However, these studies did not stratify for different SARS-CoV-2 variants and were performed before emergence of the Omicron variant and before vaccination was available. This is particularly important for patients at risk for breakthrough infections such as individuals with a compromised immune system^22-24^ and individuals who are frequently exposed to infection such as hospital workers.

Here, we surveyed neuropsychiatric symptoms after recovery from breakthrough infections with the SARS-CoV-2 Omicron variant. In a large cohort of otherwise healthy hospital workers, we assessed post-acute sequelae after SARS-CoV-2 Omicron infection by self-reported scores for fatigue, attention, mood, anxiety and somatic symptoms and found no significant differences in every scoring system in comparison with individuals without SARS-CoV-2 infection. These findings were independent of age, sex, preconditions, number of vaccinations and infections and did not correlate with time after recovery.

## Materials and methods

### Study cohort

This monocentric cross-sectional analysis was performed on a longitudinally monitored cohort of hospital workers at the University Medical Center Hamburg-Eppendorf (UKE) in Germany^25^ between February 11 2022 and 11 April 2022. In total, 1575 eligible individuals with polymerase chain reaction (PCR)-confirmed infections were contacted via email by the department of infection prevention and asked to participate in an anonymized survey after recovering from acute SARS-CoV-2 infection. We received fully completed responses from 293 individuals. Of these, we excluded individuals who reported a PCR-confirmed infection with a SARS-CoV-2 variant other than the Omicron variant (*n* = 22), those who did not state the virus variant they were infected with and were infected before January 2022 where Omicron was not yet the predominant variant (*n* = 3) and those who were hospitalized during infection (*n* = 5). Subvariants of Omicron were not reported. We included 22 patients who were infected twice with SARS-CoV-2 of which at least one infection was with the Omicron variant. In total, 263 individuals were included after recovery from acute infection (175 less than 4 weeks after positive testing and 88 more than 4 weeks after positive testing). As a control group, we included 87 randomly selected hospital workers who did not have positive SARS-CoV-2 PCR results in their medical history and reported not having been infected before. In total, we included 350 individuals (242 females and 108 males) for the final analysis.

### Assessment tools

The online questionnaires were distributed with the openly available tool *limesurvey.org*. The Fatigue Assessment Scale (FAS) score is a 10-item self-reported questionnaire.^26,27^ It measures fatigue independent of depression and was validated multiple times in the general population, the working population and patients with chronic inflammatory diseases^27^ as well as cancer.^28^ A cut-off score above 21 points indicates fatigue. The Mindful Attention Awareness Scale (MAAS) was used to assess attention in our study cohort.^29^ It is a 15-item questionnaire that measures the frequency of mindful states in day-to-day life, using both general and situation-specific statements. Higher scores in the MAAS indicate higher attention.^30^ The Patient Health Questionnaire 2 (PHQ-2) addresses the two core criteria of depressive disorders: the loss of interest and feeling depressed. The PHQ-2 has been reported to have a high sensitivity for depressive disorders and detects changes with a performance like other longer depression scales.^31^ The Generalized Anxiety Disorder 7 (GAD-7) is a well-established 7-item questionnaire to screen for anxiety severity. The score has been validated for the four most common anxiety disorders in clinical practice: generalized anxiety, panic, social anxiety and post-traumatic stress disorder. Sum scores range from 0 to 21, and a score above 10 is an indicator of the presence of an anxiety disorder.^32,33^ For the self-reported burden of somatic symptoms, we applied the Somatic Symptom Scale 8 (SSS-8), which addresses somatic symptoms falling into one of the four following groups: cardiopulmonary, gastrointestinal, pain and general.^34^ According to the score reached in the SSS-8, individuals can be categorized into five groups of somatic symptom burden: no to minimal (0–3 points), low (4–7 points), medium (8–11 points), high (12–15 points) and very high (16–32 points). The questionnaires are provided in the Appendix I.

### SARS-CoV-2 diagnostic procedures

All employees of the UKE, a tertiary care centre, were screened twice weekly for SARS-CoV-2 infection by reverse transcription PCR (RT-PCR) of nasopharyngeal swabs or gargle samples throughout the COVID-19 pandemic to prevent nosocomial transmission to coworkers and vulnerable patients. Thus, also asymptomatic and oligosymptomatic infections were detected with high sensitivity. Only study participants who reported a SARS-CoV-2 infection diagnosed by RT-PCR were included in the study.

### Statistical analysis

All statistical analyses were performed using the R environment (version 4.0.3). Difference in age and sex distribution as well as number of pre-existing conditions and vaccination status between groups were calculated by chi-square test. To assess the impact of Omicron infection less and more than 4 weeks after positive PCR on self-reported somatic and neuropsychiatric symptoms, we fitted a linear regression model with the sum score as dependent and recovery from Omicron infection (less versus more than 4 weeks) as independent variables. We controlled for sex (male and female), age groups (<30, 30–40, 40–50 and >50 years), vaccination status and the number of pre-existing conditions as independent variables. To test the interaction of independent variables with recovery from Omicron SARS-CoV-2, we performed one-way ANCOVA. We corrected for multiple comparisons by false discovery rate (FDR) adjustment. Correlation analysis between time from recovery and number of vaccinations with the sums of each score were performed using Pearson’s correlation. To analyse the minimum mean difference between groups that could have been missed by each questionnaire, we calculated the effect size using a power of 0.8, a significance level of 0.05 and the respective group sizes and multiplied it with the pooled standard deviation.

### Ethical approval

The study was approved by the ‘Ärztekammer Hamburg’ with the registration number 2022-300157-WF.

### Patient consent

All individuals gave consent for participation, data analysis and publication.

## Results

### Recruitment and study cohort

**Figure 1:**
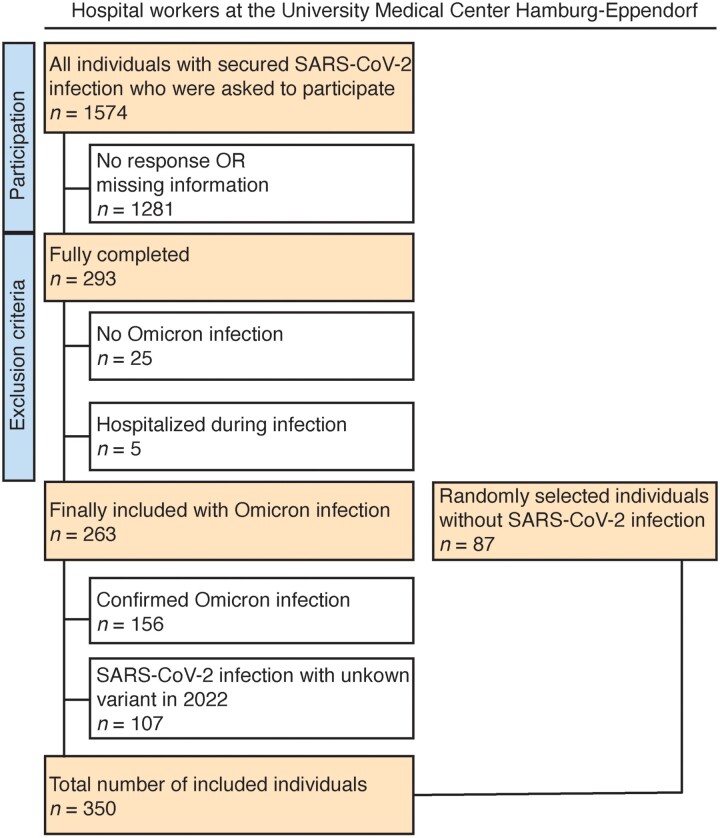
Flowchart of inclusion criteria and final cohort.

We aimed to survey neuropsychiatric and somatic disease burden in an unbiased and self-reported fashion after infection with the SARS-CoV-2 Omicron variant in a cohort of highly exposed individuals who are critical to the healthcare system.^1^ To do so, we conducted a monocentric study and made use of a representative cohort of hospital workers with predominantly up-to-date vaccination status after proven infection that mainly occurred as breakthrough infection. All participants underwent regular PCR testing for SARS-CoV-2 that ensured also the detection of asymptomatically infected individuals.^35-37^ The algorithm of inclusion and exclusion criteria is depicted in Fig. 1. In total, we included 350 individuals aged between 18 and 69 years. A total of 263 participants had a PCR-confirmed infection with SARS-CoV-2. Of these, 156 individuals had an infection with the Omicron variant. A total of 107 individuals did not report their virus variant but were infected in 2022 when COVID-19 in Germany was almost exclusively caused by the Omicron variant (www.rki.de). Thus, they were assumed to be infected with the Omicron variant. In addition, we randomly recruited 87 hospital workers as controls who have not been infected with SARS-CoV-2.

For further analysis, we stratified participants into three groups: individuals with recovery from acute infection who were included less than 4 weeks after positive PCR testing (female, *n* = 126; male, *n* = 49; 72% female; mean duration after infection = 25.6 ± 10.6 days), participants in the post-acute phase over 4 weeks after positive testing for SARS-CoV-2 (female, *n* = 63; male, *n* = 25; 72% female; mean duration after infection = 75.5 ± 37.7 days) and uninfected participants (53 female, 34 male, 61% female). In all groups, female participation was higher than male participation. All three groups had a similar age distribution (*χ*^2^ = 0.05, *P* = 1), sex distribution *χ*^2^ = 0.04, *P* = 0.98) and number of pre-existing conditions (*χ*^2^ = 0.03, *P* = 1) and were mostly vaccinated three times *χ*^2^ = 0.09, *P* = 1). Characteristics of included participants are summarized in Table 1. Together, the 350 included study participants were predominantly healthy hospital workers with almost no preconditions and mostly up-to-date vaccination status.

**Table 1:** Patient characteristics

	Characteristics	*N* total	Not infected	<4 weeks after recovery	>4 weeks after recovery
Sex *N* (%)	Female	242 (69.1)	53 (21.9)	126 (52.1)	63 (26.0)
	Male	108 (30.9)	34 (31.5)	49 (45.4)	25 (23.1)
Age (years) *N* (%)	<30	153 (43.7)	36 (23.5)	86 (56.2)	31 (20.3)
	30–39	94 (26.9)	21(22.3)	47 (50.0)	26 (27.7)
	40–49	66 (18.9)	19 (28.8)	27 (40.9)	20 (30.3)
	>50	37 (10.5)	11 (29.7)	15 (40.6)	11 (29.7)
Vaccinations *N* (%)	0	11(3.1)	3 (27.3)	3 (27.3)	5 (45.4)
	1	4 (1.1)	0 (0)	2 (50.0)	2 (50.0)
	2	22 (6.3)	2 (9.1)	14 (63.6)	6 (27.3)
	3	310 (88.6)	82 (26.5)	154 (49.7)	74 (23.8)
	4	3 (0.9)	0 (0)	2 (66.6)	1 (33.4)
Pre-existing conditions *N* (%)	0	239 (68.3)	62 (25.9)	119 (49.8)	58 (24.3)
	1	74 (21.1)	17 (23.0)	39 (52.7)	18 (24.3)
	2	25 (7.1)	5 (20.0)	13 (52.0)	7 (28.0)
	3	10 (2.9)	3 (30.0)	3 (30.0)	4 (40.0)
	4	2 (0.6)	0 (0)	1 (50.0)	1 (50.0)
SARS-CoV-2 *N* (%)	Confirmed Omicron by PCR	156 (44.6)	NA	99 (63.5)	57 (36.5)
	Infection in 2022	107 (30.6)	NA	76 (71.0)	31 (29.0)
	Not infected	87 (24.8)	NA	NA	NA

### Assessing neuropsychiatric sequelae

**Figure 2:**
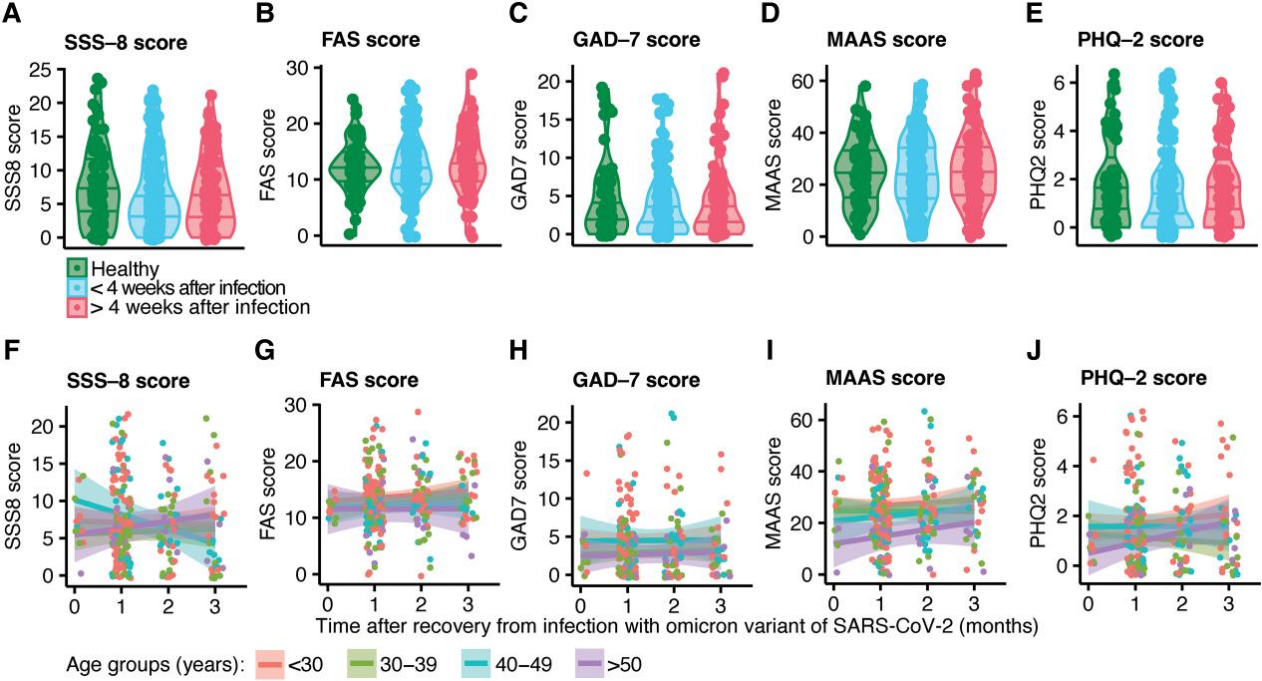
**No differences in self-reported somatic and neuropsychiatric deficits after recovery from Omicron.** (**A–E**) Comparison of SSS-8 (**A**; healthy versus <4 weeks *P* = 0.27, healthy versus >4 weeks *P* = 0.091), FAS (**B**; healthy versus <4 weeks *P* = 0.76, healthy versus >4 weeks *P* = 0.69), GAD-7 (**C**; healthy versus <4 weeks *P* = 0.46, healthy versus >4 weeks *P* = 0.9), MAAS (**D**; healthy versus <4 weeks *P* = 0.2, healthy versus >4 weeks *P* = 0.59), PHQ-2 (**E**; healthy versus <4 weeks *P* = 0.07, healthy versus >4 weeks *P* = 0.41) between individuals that did not contract SARS-CoV-2 infection and individuals less and more than 4 weeks after recovery from SARS-CoV-2 infection. Linear regression model with correction for sex and age was used for statistical analysis. (**F–J**) Correlation analysis of months after recovery and SSS-8 (**F**; <30 years *P* = 0.56, 30–39 years *P* = 0.74, 40–49 years *P* = 0.11, >50 years *P* = 0.38), FAS (**G**; <30 years *P* = 0.59, 30–39 years *P* = 0.23, 40–49 years *P* = 0.75, >50 years *P* = 0.98), GAD-7 (**H**; <30 years *P* = 0.89, 30–39 years *P* = 0.67, 40–49 years *P* = 0.96, >50 years *P* = 0.78), MAAS (**I**; <30 years *P* = 0.59, 30–39 years *P* = 0.97, 40–49 years *P* = 0.5, >50 years *P* = 0.29) and PHQ-2 (**J**; <30 years *P* = 0.49, 30–39 years *P* = 0.53, 40–49 years *P* = 0.99, >50 years *P* = 0.11) scores. Each data point represents an individual. Statistical analysis was performed by Pearson correlation analysis.

To systematically survey a wide range of neuropsychiatric domains with well-established tests, we assessed somatic and neuropsychiatric symptoms by conducting an online survey. This allowed us to anonymously receive an authentic insight into symptoms. First, we addressed somatic symptoms with the SSS-8.^34^ These symptoms include headache, abdominal pain, limb pain, back pain, sleep disturbances, fatigue, dizziness and shortness of breath. We did not find differences in self-reported items between participants less than 4 weeks (*P* = 0.27) and participants more than 4 weeks after recovery as well as in comparison with uninfected controls (*P* = 0.091; Fig. 2A).

Next, we quantified neuropsychiatric symptoms with a variety of established and commonly used screening questionnaires. We assessed fatigue that is reported to be one of the most common symptoms of Long COVID.^38^ By employing the Fatigue Assessment Score (FAS), we observed no significant differences between uninfected controls and individuals that recovered from infection with the SARS-CoV-2 Omicron variant that was also independent of time after infection (<4 weeks after recovery *P* = 0.69; >4 weeks after recovery *P* = 0.76). Of note, the mean FAS score for all groups was below 21 points, which is commonly used as a threshold for the diagnosis of fatigue (Fig. 2B). Subsequently, we evaluated anxiety by applying the GAD-7 questionnaire. A total score of 10 or more indicates the presence of an anxiety disorder. Again, a previous mild SARS-CoV-2 infection with the Omicron variant did not influence the scoring in the anxiety levels as assessed with the GAD-7 after recovery from acute infection in vaccinated individuals (<4 weeks *P* = 0.2; >4 weeks *P* = 0.59; Fig. 2C). Additionally, we assessed attention and awareness by including the MAAS in the survey with lower scores indicating lower levels of attention. We were not able to detect significant differences between uninfected controls and patients after recovery from mild Omicron breakthrough infection (<4 weeks *P* = 0.9; >4 weeks *P* = 0.46; Fig. 2D). We asked whether infected individuals were more likely to show signs of depression as assessed with the PHQ-2 and observed no differences between infected individuals and uninfected controls (<4 weeks after recovery *P* = 0.067; >4 weeks after recovery *P* = 0.41; Fig. 2E). The pathological threshold for each questionnaire and the mean score and standard deviation reached by each group (non-infected, <4 weeks after infection, >4 weeks after infection) are summarized in Table 2. Furthermore, for each questionnaire, we analysed the minimum mean difference between non-infected individuals and <4 weeks after infection as well as non-infected and >4 weeks after infection that would have been necessary to reach significance given our sample sizes, a power of 0.8, a significance level of 0.05 and the pooled standard deviation (Table 2).

**Table 2:** Descriptive statistics for whole cohort

Test	Mean	SD	Mean difference compared with not infected	Minimum mean difference for detection
**FAS (pathological threshold > 21)**				
Not infected	12.5	4.4	NA	NA
<4 weeks after recovery	12.7	5.0	0.2	1.5
>4 weeks after recovery	13.3	5.3	0.8	1.8
**MAAS (pathological threshold < 17)**				
Not infected	24.6	12.4	NA	NA
<4 weeks after recovery	24.1	13.4	−0.5	4.2
>4 weeks after recovery	25.7	13.6	1.2	4.9
**GAD-7 (pathological threshold > 10)**				
Not infected	4.4	4.7	NA	NA
<4 weeks after recovery	3.7	4.0	−0.7	1.4
>4 weeks after recovery	4.2	4.7	−0.2	1.8
**PHQ-2 (pathological threshold > 2)**				
Not infected	1.7	1.7	NA	NA
<4 weeks after recovery	1.4	1.6	−0.3	0.5
>4 weeks after recovery	1.6	1.6	−0.1	0.6
**SSS-8 (high burden > 11)**				
Not infected	7.5	5.9	NA	NA
<4 weeks after recovery	6.9	5.6	−0.6	1.9
>4 weeks after recovery	6.4	5.3	−1.1	2.1

In addition to acute and post-acute infection, we tested the presence of several preconditions, such as sex, age and the number of vaccinations as independent variables for each score (results are shown in Table 3). For significant associations, we calculated the interaction with recovery less and more than 4 weeks after infection. Of note, we did not find any significant interactions of SARS-CoV-2 infection with age, sex, vaccination status, the number of preconditions and the number of infections (results are shown in Table 4). Thus, the lack of differences in self-reported somatic and neuropsychiatric symptoms between uninfected controls and individuals with acute or post-acute infection is independent of age, sex, vaccination status, the number of preconditions and the number of infections.

**Table 3:** Descriptive statistics by subgroups

Variable	Estimate	SE	*t*-statistics	*P*-value	Significance
**FAS**					
<4 weeks after infection	−0.26	0.62	−0.42	0.76	ns
>4 weeks after infection	0.44	0.71	0.62	0.69	ns
Age 30–39	−0.72	0.62	−1.16	0.37	ns
Age 40–49	−1.45	0.69	−2.09	0.084	ns
Age >50	−1.39	0.87	−1.60	0.2	ns
Sex	2.49	0.55	4.50	4.1e−05	***
Preconditions	−2.34	0.54	−4.32	6.3e−05	***
Vaccination	−0.36	1.45	−0.24	0.81	Ns
**MAAS**					
<4 weeks after infection	−1.70	1.66	−1.03	0.2	ns
>4 weeks after infection	0.44	1.90	0.23	0.59	ns
Age 30–39	−0.20	1.65	−0.12	0.2	ns
Age 40–49	−4.36	1.86	−2.34	0.94	ns
Age >50	−8.76	2.33	−3.76	0.24	ns
Sex (female)	4.62	1.48	3.12	0.17	ns
Preconditions	−5.75	1.45	−3.96	4.7e−06	***
Vaccination	−1.36	3.89	−0.35	0.59	ns
**GAD-7**					
<4 weeks after infection	−0.90	0.56	−1.61	0.46	ns
>4 weeks after infection	−0.45	0.64	−0.70	0.9	ns
Age 30–39	−0.92	0.56	−1.65	0.9	ns
Age 40–49	−0.05	0.63	−0.07	0.036	*
Age >50	−1.11	0.78	−1.42	0.00061	***
Sex (female)	0.95	0.50	1.92	0.0044	**
Preconditions	−2.50	0.49	−5.12	0.00041	***
Vaccination	−0.83	1.31	−0.63	0.9	ns
**PHQ-2**					
<4 weeks after infection	−0.42	0.21	−2.02	0.067	ns
>4 weeks after infection	−0.20	0.24	−0.83	0.41	ns
Age 30–39	−0.51	0.21	−2.46	0.032	*
Age 40–49	−0.22	0.23	−0.94	0.39	ns
Age >50	−0.60	0.29	−2.04	0.067	ns
Sex (female)	0.67	0.19	3.60	0.0016	**
Preconditions	−0.58	0.18	−3.19	0.0046	**
Vaccination	−0.62	0.49	−1.26	0.27	ns
**SSS-8**					
<4 weeks after infection	−0.93	0.69	−1.35	0.27	ns
>4 weeks after infection	−1.63	0.80	−2.05	0.091	ns
Age 30–39	−0.49	0.69	−0.70	0.62	ns
Age 40–49	0.23	0.78	0.30	0.86	ns
Age >50	0.05	0.98	0.05	0.96	ns
Sex (female)	2.88	0.62	4.65	1.5e−05	***
Preconditions	−3.31	0.61	−5.45	4.3e−07	***
Vaccination	−3.20	1.63	−1.96	0.091	ns

**Table 4:** Summary statistics

Variable	*F*-statistic	*P*-value	Significance
**FAS**			
Sex	2.57	0.8	ns
Age	0.21	0.97	ns
Preconditions	0.60	0.93	ns
Vaccination	0.33	0.93	ns
**GAD-7**			
Sex	0.31	0.93	ns
Age	0.68	0.93	ns
Preconditions	0.35	0.93	ns
Vaccination	0.46	0.93	ns
**MAAS**			
Sex	1.04	0.93	ns
Age	0.91	0.93	ns
Preconditions	0.54	0.93	ns
Vaccination	0.41	0.93	ns
**PHQ-2**			
Sex	0.17	0.94	ns
Age	0.39	0.94	ns
Preconditions	0.56	0.93	ns
Vaccination	2.14	0.8	ns
**SSS-8**			
Sex	1.68	0.93	ns
Age	0.47	0.94	ns
Preconditions	3.67	0.6	ns
Vaccination	0.51	0.93	ns

Since we observed significant differences in some of the scores between different age groups (Table 5), we performed correlation analyses of each score and time after recovery for all 263 infected individuals separated by age groups to rule out that certain age groups were affected by neuropsychiatric symptoms following SARS-CoV-2 infection while others were not. In accordance with our other findings, we did not observe significant correlations between time after infection and persistent somatic symptoms (Fig. 2F–J) and neuropsychiatric deficits for each score (results are shown in Table 5). Furthermore, we tested for correlation between persistent symptoms and number of vaccinations, which was again not significant for each age group (results are shown in Table 5). We analysed the impact of re-infections on persistent symptoms by correlation analysis and detected no significant differences in both sexes (FAS: female, *P* = 0.10, *R* = −0.11; male, *P* = 0.30, *R* = −0.10; GAD-7: female, *P* = 0.92, *R* = 0.006; male, *P* = 0.09, *R* = −0.16; MAAS: female, *P* = 0.34, *R* = 0.06; male, *P* = 0.21, *R* = −0.12; PHQ-2: female, *P* = 0.62, *R* = −0.03; male, *P* = 0.07, *R* = −0.17; SSS-8: female, *P* = 0.07, *R* = −0.12; male, *P* = 0.55, *R* = −0.06). Last, we compared patients with one infection (*n* = 241) and re-infection (*n* = 22) and identified no significant differences in remaining somatic (SSS-8, *P* = 0.65) or neuropsychiatric symptoms (FAS, *P* = 0.40; GAD-7, *P* = 0.71; MAAS, *P* = 0.88; PHQ-2, *P* = 0.90) underlining the absence of post-acute neuropsychiatric deficits in individuals with up-to-date vaccination status after recovery from Omicron infection.

**Table 5:** Correlation analysis with sum scores and time after recovery and number of vaccinations

	Time after recovery	Number of vaccinations
**FAS**		
<30 years	0.05	−0.13
30–39 years	0.14	0.09
40–49 years	0.05	−0.03
>50 years	0.004	−0.03
**GAD–7**		
<30 years	0.01	−0.13
30–39 years	0.05	−0.02
40–49 years	0.01	−0.07
>50 years	0.06	0.05
**MAAS**		
<30 years	0.05	−0.07
30–39 years	−0.004	0.03
40–49 years	0.1	0.19
>50 years	0.22	−0.16
**PHQ-2**		
<30 years	0.06	−0.08
30–39 years	−0.08	−0.12
40–49 years	0.001	0.006
>50 years	0.32	−0.2
**SSS–8**		
<30 years	−0.05	−0.09
30–39 years	0.04	0.04
40–49 years	−0.24	−0.21
>50 years	0.18	−0.17

## Discussion

In this cross-sectional single-centre study, we investigated self-reported neuropsychiatric and somatic symptoms in individuals who experienced a mild breakthrough infection with the SARS-CoV-2 variant Omicron as compared with uninfected controls in a cohort of German hospital workers mostly having an up-to-date vaccination status. We used a comprehensive online questionnaire, addressing common neuropsychiatric symptoms including fatigue, attention deficits, depression and anxiety as well as the self-reported burden of somatic symptoms. For these symptoms, we did not detect any significant differences between individuals less and more than 4 weeks after infection with SARS-CoV-2 and uninfected controls after adjustment for sex, age, number of preconditions and vaccination against SARS-CoV-2.

Our results are in contrast to previous studies that report frequent and severe post-acute sequelae of COVID-19 during the first phases of the pandemic.^3,39,40^ Recent meta-analyses and systematic reviews revealed that more than half of individuals experienced at least one symptom after recovery from SARS-CoV-2 infection that persisted for more than 6 months with mobility impairment, pulmonary abnormalities and mental health disorders being the most common.^40^ Furthermore, several publications showed that neuropsychiatric deficits occur independently of the severity of the initial infection.^9,18,40^ However, these studies were conducted at a time when mainly the Wild-Type, Alpha, Beta and Delta variants of SARS-CoV-2 were prevalent, vaccination was not available, and Omicron had not yet emerged. In contrast, cross-sectional studies that correlated cerebral imaging with neuropsychological deficits reported no differences associated with the Wild-Type variant during the first wave.^41^ Clinical characterization of individuals after recovery from infection with Omicron is of utmost importance since it became the predominant variant worldwide due to high infectivity and poor cross-reactivity of available vaccinations.^42,43^ It is unclear, whether the Omicron variant leads to persistent or novel neuropsychiatric and somatic symptoms after recovery from acute infection. Here, we demonstrate that vaccinated hospital workers after recovery from mild infection with Omicron do not have a higher burden of symptoms than hospital workers who never experienced SARS-CoV-2 infection. This is further supported by a recent study that analysed data from the COVID Symptoms Study app in the UK and showed that the risk of developing Long COVID was significantly lower in individuals who were infected with the Omicron variant compared with those infected with the Delta variant.^44^

The mechanistic underpinnings of Long COVID after infection with the Wild-Type, Alpha, Beta and Delta variants remain unclear. Neuropsychiatric deficits during the acute infection might be on the one hand explained by SARS-CoV-2–induced dysregulation of multiple organ systems^45^ and on the other hand by direct invasion of the central nervous system (CNS).^46,47^ Although *in vitro* studies suggested neuronal tropism of SARS-CoV-2, post-mortem analyses of large cohorts could only identify SARS-CoV-2 by PCR or immunohistochemical staining of its capsid protein in the blood vessels and endothelial cells in the CNS,^48,49^ together with bystander immune cell activation^50,51^ but without parenchymal infiltration or neuronal infection. Additionally, there is no evidence of long-lasting persistence of SARS-CoV-2 in the CNS. On a systems level, elevated concentrations of the cytokine CCL11 were detected in individuals suffering from Long COVID as compared with those who experienced no symptoms following infection. Subsequent studies in mice suggest that CCL11 leads to white matter enriched microglial reactivity followed by a dysregulation of the oligodendroglial lineage and myelin loss.^19^ Other hypotheses include microclot formation in the blood^52^ and immunological dysfunctions following infection with a lack of naïve B and T cells as well as elevated expression of type I and III interferons.^53^ However, these studies did not analyse infection with the Omicron variant and lack specificity for SARS-CoV-2 since individuals after recovery from infection with other common viruses such as influenza virus were not investigated. Furthermore, we consider it important to include individuals who have not been infected as controls since the neuropsychiatric and somatic symptoms that are associated with Long COVID are unspecific, highly prevalent in the general population and could be exacerbated by the pandemic itself with its fundamental impact on daily living.^54^ Furthermore, Long COVID is currently a constant topic in the media that could contribute to overestimation of its prevalence and foster heightened attention in infected individuals.^55^ Studies as ours might therefore help mitigate negative expectations in susceptible individuals.

Our study is limited by its cross-sectional monocentric design without longitudinal follow-up and limited sample size. We therefore calculated the difference in means for each questionnaire that could have been undetected using our sample size. However, we included a healthy control group without SARS-CoV-2 infection and did not identify differences in comparison with individuals after recovery from infection. Our study focused on hospital workers, potentially leading to selection bias since this cohort might be specifically vulnerable to develop persistent symptoms of SARS-CoV-2 infection due to the heightened workload and pressure of medical staff resulting from the pandemic and increased attention to symptoms. However, both infected and uninfected individuals reached scores mostly within the physiological range for all questionnaires. One possible explanation is the inclusion of asymptomatic infections and the better position of hospital workers to mitigate symptoms. Furthermore, we analysed self-reported symptoms, which were not affirmed through clinical examination or diagnostic tools by healthcare professionals and are potentially prone to selection bias. However, one advantage of this approach is that it facilitates the investigation of symptoms and disease burden in a large number of individuals in an unbiased and cost-effective manner. Thus, this approach has the potential to perform population-based screens to assess prevalence of post-acute sequelae. For the future, standardized questionnaires to quantify persistent neuropsychiatric symptoms after infection with SARS-CoV-2 would be desirable. Since most of the participants were vaccinated at the time of the survey, it is difficult to discriminate whether the absence of self-reported post-acute neuropsychiatric and somatic symptoms is due to vaccination or if the Omicron variant of SARS-CoV-2 is less likely to cause these symptoms. The latter is supported by a large cohort study where vaccination showed no effect on post-COVID fatigue, cognitive symptoms or affective disorders before the emergence of the Omicron variant.^56^ In our study, we did not discriminate between Omicron subvariants. This should be systematically investigated by future studies due to the high escape of especially the BA.5 subvariant against neutralizing antibodies^57^ that might result in higher acute and persistent disease burden. Our cross-sectional study was restricted to hospital workers, and we cannot exclude a population bias. Nonetheless, our findings are supported by other studies that compared self-reported symptoms after infection with the Omicron and Delta variants and reported fewer individuals with long-lasting symptoms after Omicron infection.^44^ Furthermore, we did not have serological data for our control group available, and they were included due to self-reportedly denying past infection and the absence of positive SARS-CoV-2 PCRs in their medical history. The close monitoring of SARS-CoV-2 positivity by PCR in our cohort enabled us to sensitively identify individuals with mild disease or asymptomatic infection.

## Conclusion

In summary, our data allow us to conclude that vaccinated hospital workers after recovery from mild infection with the Omicron variant of SARS-CoV-2 do not suffer from persistent somatic and neuropsychiatric symptoms in our study cohort. These findings have wide clinical implications and will guide occupational health specialists and other public health specialists in the management of potential Long COVID patients after recovery from Omicron infection.

## Data Availability

Data are available from the corresponding author, upon reasonable request. Data are not publicly available due to ethical restrictions because the information contained in those data could compromise the privacy of the reported patients.

## Appendix I.

Online questionnaires.

How old are you?

<20

20–29

30–39

40–49

50–59

60–69

>69

What is your gender?

Female

Male

Diverse

Do you suffer from any pre-existing medical conditions? Please select all that apply.

Heart attack

Heart failure

Peripheral artery disease (PAD)

Chronic obstructive pulmonary disease (COPD)

Asthma bronchiale

Gastric or duodenal ulcer

Mild liver disease

Moderate to severe liver disease

Moderate to severe renal failure

Collagenosis

Diabetes mellitus without end organ damage

Diabetes mellitus with end-organ damage

Stroke or transient ischaemic attack (TIA)

Hemiplegia

Multiple sclerosis

Migraine

Other headache disorder

Tumour disease (non-metastatic)

Tumour disease (metastasized)

Leukaemia

Lymphoma

AIDS

Dementia

Depression

Anxiety disorder

Other previous psychiatric illness

Other chronic infectious disease

Other autoimmune disease

How many COVID-19 vaccine doses did you receive to date?

0

1

2

3

4

When did you receive your first COVID-19 vaccination?

Month/year

Which COVID-19 vaccine did you receive for the first dose?

BioNTech/Pfizer

Moderna

AstraZeneca

Johnson & Johnson

I do not know

Other

When did you receive your second COVID-19 vaccination?

Month/year

Which COVID-19 vaccine did you receive for the second dose?

BioNTech/Pfizer

Moderna

AstraZeneca

Johnson & Johnson

I do not know

Other

When did you receive your third COVID-19 vaccination?

Month/year

Which COVID-19 vaccine did you receive for the third dose?

BioNTech/Pfizer

Moderna

AstraZeneca

Johnson & Johnson

I do not know

Other

When did you receive your fourth COVID-19 vaccination?

Month/year

Which COVID-19 vaccine did you receive for the fourth dose?

BioNTech/Pfizer

Moderna

AstraZeneca

Johnson & Johnson

I do not know

Other

Did you have contract or more SARS-CoV-2 infections diagnosed by PCR in the past?

No

Yes, one infection

Yes, two infections

Yes, three infections

When did you contract the first SARS-CoV-2 infection?

Month/year

How many COVID-19 vaccine doses had you received at the time of your first SARS-CoV-2 infection?

None

1

2

3

4

With which variant did you become infected at the time of your first SARS-CoV-2 infection?

Wild Type

Alpha

Beta

Gamma

Delta

Omicron

Not known

Did you require inpatient medical treatment at the time of your first SARS-CoV-2 infection?

Yes

No

Did you require intensive care treatment at the time of your first SARS-CoV-2 infection?

Yes

No

When did you contract the second SARS-CoV-2 infection?

Month/year

How many COVID-19 vaccine doses had you received at the time of your second SARS-CoV-2 infection?

None

1

2

3

4

With which variant did you become infected at the time of your second SARS-CoV-2 infection?

Wild Type

Alpha

Beta

Gamma

Delta

Omicron

Not known

Did you require inpatient medical treatment at the time of your second SARS-CoV-2 infection?

Yes

No

Did you require intensive care treatment at the time of your second SARS-CoV-2 infection?

Yes

No

When did you contract the third SARS-CoV-2 infection?

Month/year

How many COVID-19 vaccine doses had you received at the time of third SARS-CoV-2 infection?

None

1

2

3

4

With which variant did you become infected at the time of your third SARS-CoV-2 infection?

Wild Type

Alpha

Beta

Gamma

Delta

Omicron

Not known

Did you require inpatient medical treatment at the time of your third SARS-CoV-2 infection?

Yes

No

Did you require intensive care treatment at the time of your third SARS-CoV-2 infection?

Yes

No

During the past 7 days, how much have you been bothered by any of the following problems?

(not at all; a little bit; somewhat; quite a bit; very much)

Stomach or bowel problems

Back pain

Pain in your arms, legs or joints

Headaches

Chest pain or shortness of breath

Dizziness

Feeling tired or having low energy

Trouble sleeping

Over the last 2 weeks, how often have you been bothered by any of the following problems?

(not at all; several days; more than half the days; nearly every day)

Little interest or pleasure in doing things

Feeling down, depressed or hopeless

Feeling nervous, anxious or on edge?

Not being able to stop or control worrying?

Worrying too much about different things?

Trouble relaxing?

Being so restless that it is hard to sit still?

Becoming easily annoyed or irritable?

Feeling afraid as if something awful might happen?

The following ten statements refer to how you usually feel. Per statement you can choose one out of five answer categories, varying from never to always.

(never; sometimes/about monthly or less; regularly/about a few times a month; often/about weekly; always/about every day)

I am bothered by fatigue

I get tired very quickly

I don’t do much during the day

I have enough energy for everyday life

Physically, I feel exhausted

I have problems to start things

I have problems to think clearly

I feel no desire to do anything

Mentally, I feel exhausted

When I am doing something, I can concentrate quite well

Below is a collection of statements about your everyday experience. Using the 1–6 scale below, please indicate how frequently or infrequently you currently have each experience. Please answer according to what really reflects your experience rather than what you think your experience should be. Please treat each item separately from every other item.

(1, almost always; 2, very frequently; 3, somewhat frequently; 4, somewhat infrequently; 5, very infrequently; 6, almost never)

I could be experiencing some emotion and not be conscious of it until some time later.

I break or spill things because of carelessness, not paying attention or thinking of something else.

I find it difficult to stay focused on what’s happening in the present.

I tend to walk quickly to get where I’m going without paying attention to what I experience along the way.

I tend not to notice feelings of physical tension or discomfort until they really grab my attention.

I forget a person’s name almost as soon as I’ve been told it for the first time.

It seems I am ‘running on automatic’, without much awareness of what I’m doing.

I rush through activities without being really attentive to them.

I get so focused on the goal I want to achieve that I lose touch with what I’m doing right now to get there.

I do jobs or tasks automatically, without being aware of what I’m doing.

I find myself listening to someone with one ear, doing something else at the same time.

I drive places on ‘automatic pilot’ and then wonder why I went there.

I find myself preoccupied with the future or the past.

I find myself doing things without paying attention.

I snack without being aware that I’m eating.

## Abbreviations

COVID-19 =: coronavirus SARS-CoV-2
FAS =: Fatigue Assessment Scale
FDR =: false discovery rate
GAD-7 =: Generalized Anxiety Disorder 7
MAAS =: Mindful Attention Awareness Scale
PHQ-2 =: Patient Health Questionnaire 2
RT-PCR =: reverse transcription polymerase chain reaction
SARS-CoV-2 =: severe acute respiratory syndrome coronavirus 2
SSS-8 =: Somatic Symptom Scale 8
UKE =: University Medical Center Hamburg-Eppendorf
